# Dissipative Systems Driven by the Decarboxylation of Activated Carboxylic Acids

**DOI:** 10.1021/acs.accounts.3c00047

**Published:** 2023-03-14

**Authors:** Daniele Del Giudice, Stefano Di Stefano

**Affiliations:** Dipartimento di Chimica and ISB-CNR Sede Secondaria di Roma - Meccanismi di Reazione, Università degli Studi di Roma “La Sapienza”, P.le A. Moro 5, 00185 Rome, Italy

## Abstract

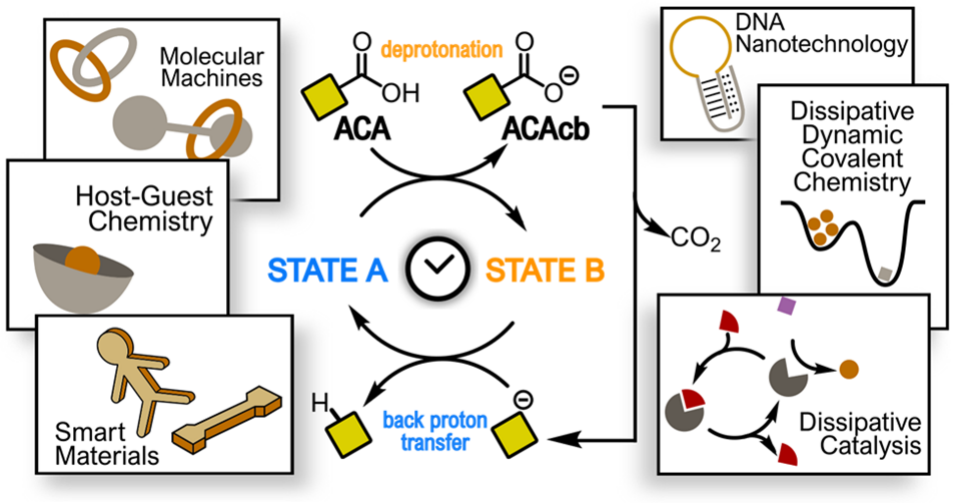

The achievement of artificial systems capable of being maintained in out-of-equilibrium states featuring functional properties is a main goal of current chemical research. Absorption of electromagnetic radiation or consumption of a chemical species (a “chemical fuel”) are the two strategies typically employed to reach such out-of-equilibrium states, which have to persist as long as one of the above stimuli is present. For this reason such systems are often referred to as “dissipative systems”. In the simplest scheme, the dissipative system is initially found in a resting, equilibrium state. The addition of a chemical fuel causes the system to shift to an out-of-equilibrium state. When the fuel is exhausted, the system reverts to the initial, equilibrium state. Thus, from a mechanistic standpoint, the dissipative system turns out to be a catalyst for the fuel consumption. It has to be noted that, although very simple, this scheme implies the chance to temporally control the dissipative system. In principle, modulating the nature and/or the amount of the chemical fuel added, one can have full control of the time spent by the system in the out-of-equilibrium state.

In 2016, we found that 2-cyano-2-phenylpropanoic acid (**1a**), whose decarboxylation proceeds smoothly under mild basic conditions, could be used as a chemical fuel to drive the back and forth motion of a catenane-based molecular switch. The acid donates a proton to the catenane that passes from the neutral state A to the transient protonated state B. Decarboxylation of the resulting carboxylate (**1a**cb), generates a carbanion, which, being a strong base, retakes the proton from the protonated catenane that, consequently, returns to the initial state A. The larger the amount of the added fuel, the longer the time spent by the catenane in the transient, out-of-equilibrium state. Since then, acid **1a** and other activated carboxylic acids (ACAs) have been used to drive the operation of a large number of dissipative systems based on the acid–base reaction, from molecular machines to host–guest systems, from catalysts to smart materials, and so on. This Account illustrates such systems with the purpose to show the wide applicability of ACAs as chemical fuels. This generality is due to the simplicity of the idea underlying the operation principle of ACAs, which always translates into simple experimental requirements.

## Key References

- BerrocalJ. A.; BiaginiC.; MandoliniL.; Di StefanoS.Coupling of the Decarboxylation of 2-Cyano-2-phenylpropanoic Acid to Large-Amplitude Motions: A Convenient Fuel for an Acid–Base-Operated Molecular Switch. Angew. Chem., Int. Ed.2016, 55, 6997–700110.1002/anie.20160259427145060.^1^*This was the first report in which an activated carboxylic acid (ACA), namely, 2-cyano-2-phenylpropanoic acid, was used to drive the operation of a dissipative system, in this case a catenane based molecular switch.*
- BiaginiC.; AlbanoS.; CarusoR.; MandoliniL.; BerrocalJ. A.; Di StefanoS.Variations in the Fuel Structure Control the Rate of the Back and Forth Motions of a Chemically Fuelled Molecular Switch. Chem. Sci.2018, 9, 181–1882962908610.1039/c7sc04123cPMC5869305.^2^*In this article, it was shown that manipulating the chemical structure of the fuel, it was possible to temporally control the duration of the out-of-equilibrium state of a dissipative system.*
- MariottiniD.; Del GiudiceD.; ErcolaniG.; Di StefanoS.; RicciF.Dissipative Operation of pH-responsive DNA-based Nanodevices. Chem. Sci.2021, 12, 11735–117393465970910.1039/d1sc03435aPMC8442697.^3^*This article showed that ACA fuels can be used to temporally drive the interactions between a DNA-based receptor/cargo couple in water solution.*
- Del GiudiceD.; ValentiniM.; MelchiorreG.; SpatolaE.; Di StefanoS.Dissipative Dynamic Covalent Chemistry (DDCvC) Based on the Transimination Reaction. Chem.—Eur. J.2022, 28, e2022006853526299210.1002/chem.202200685.^4^*Here ACA fuels were exploited to bring dynamic libraries of imines out-of-equilibrium. At the addition of the fuel, the relative concentrations of the library members were transitorily changed, and the new composition persisted as long as the fuel was present.*

## Introduction

Abiotic systems which take advantage of chemical “fuels”^5,6^ (chemical reactants) to be maintained in functional, out-of-equilibrium^7^ states represent a hot topic in current chemical research.^8^ Such interest is mainly motivated by the prospect of designing complex molecular structures and materials with time-programmable properties.^8^ The systems whose operation requires the consumption of chemical fuels are often defined as “dissipative”.^9^ Throughout this Account, we will use the term dissipative to indicate a system whose out-of-equilibrium state^7^ is maintained by fuel consumption; in other words, a system constituted by a catalyst for the conversion of a fuel into waste, which therefore is able to transitorily divert the free energy associated with the consumption of a fuel toward valuable processes (system operation).^10^

Many of the dissipative artificial systems reported so far are driven by bioinspired fuels such as nucleoside triphosphates (ATP and the like) or fragments of RNA/DNA and often take advantage of the presence of enzymes for their operation.^11^ However, a number of abiotic fuels have been also used to drive man-made dissipative systems. The operation mechanisms of such abiotic fuels are generally simpler, not requiring the presence of enzymes or narrow experimental conditions. Remarkable examples of abiotic fuels are condensing agents like EDC (1-ethyl-3-(3-(dimethylamino)propyl)carbodiimide) or protecting ones like Fmoc-Cl (9-fluorenylmethyloxycarbonyl chloride), which have been applied in the field of dissipative self-assembly and molecular machines.^6,12,13^

Another class of frequently applied abiotic fuels, to which this Account is devoted, is that of activated carboxylic acids (ACAs) prone to give base-promoted decarboxylation reactions. Such reagents proved to be ideal fuels for the operation of dissipative systems based on the acid–base reaction, including molecular switches and motors, host–guest systems, DNA-based receptors, dynamic libraries, catalysts, and smart materials (*vide infra*). As it will be shown in the next pages, the wide applicability of ACAs is due to the simplicity of their operation mechanism.

## General Concepts

**Figure 1:**
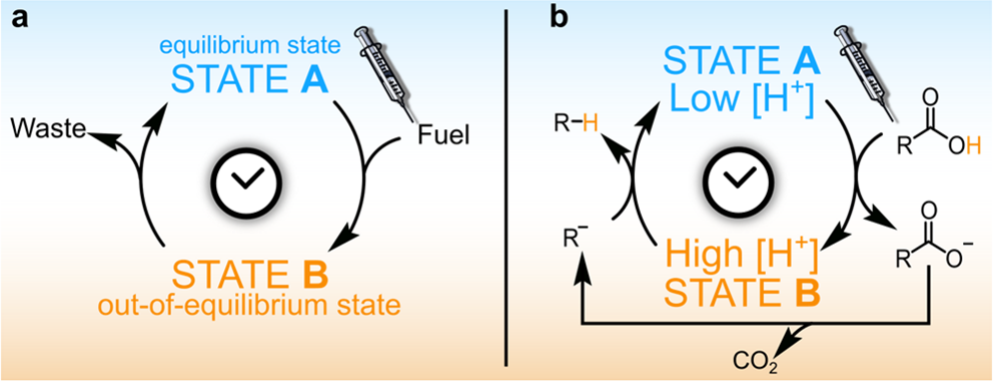
(a) Operation of a generic dissipative system. (b) An ACA-driven dissipative system.

Figure 1a shows a simplified scheme for the operation of a fuel-driven dissipative system, which is initially in its resting, equilibrium state A. After the addition of a fuel, the system is induced to pass from state A to the new state B, which is an out-of-equilibrium state. Indeed, the system persists in state B only as long as the fuel is present. When the latter is exhausted, the system goes back to the initial, resting state A. As stated before, the system just acts as a catalyst for fuel consumption.

A peculiar feature of dissipative systems resides in the opportunity to temporally control the duration of state B, which translates into time-control of their structural and chemical–physical properties.

ACA fuels are used when the dissipative system is based on the acid–base reaction (see Figure 1b). In this case, the system possesses a basic site which is protonated by the ACA. Consequently, it passes from the neutral, resting state A to the protonated, out-of-equilibrium^7^ state B. Then, decarboxylation of the conjugate base of the ACA (ACAcb, cb denotes “conjugate base”) takes place, and the corresponding carbanion is formed. The latter is a strong base able to retake the proton from the protonated system, which reverts to the initial state A. Remarkably, the duration of the out-of-equilibrium state B, can be controlled by varying the chemical nature of the ACA, which influences the rate of the decarboxylation or back proton transfer step and/or its amount. Indeed, the higher the quantity of the added fuel, the longer the time required for its consumption, the longer the duration of state B.

## The ACA Family

**Figure 2:**
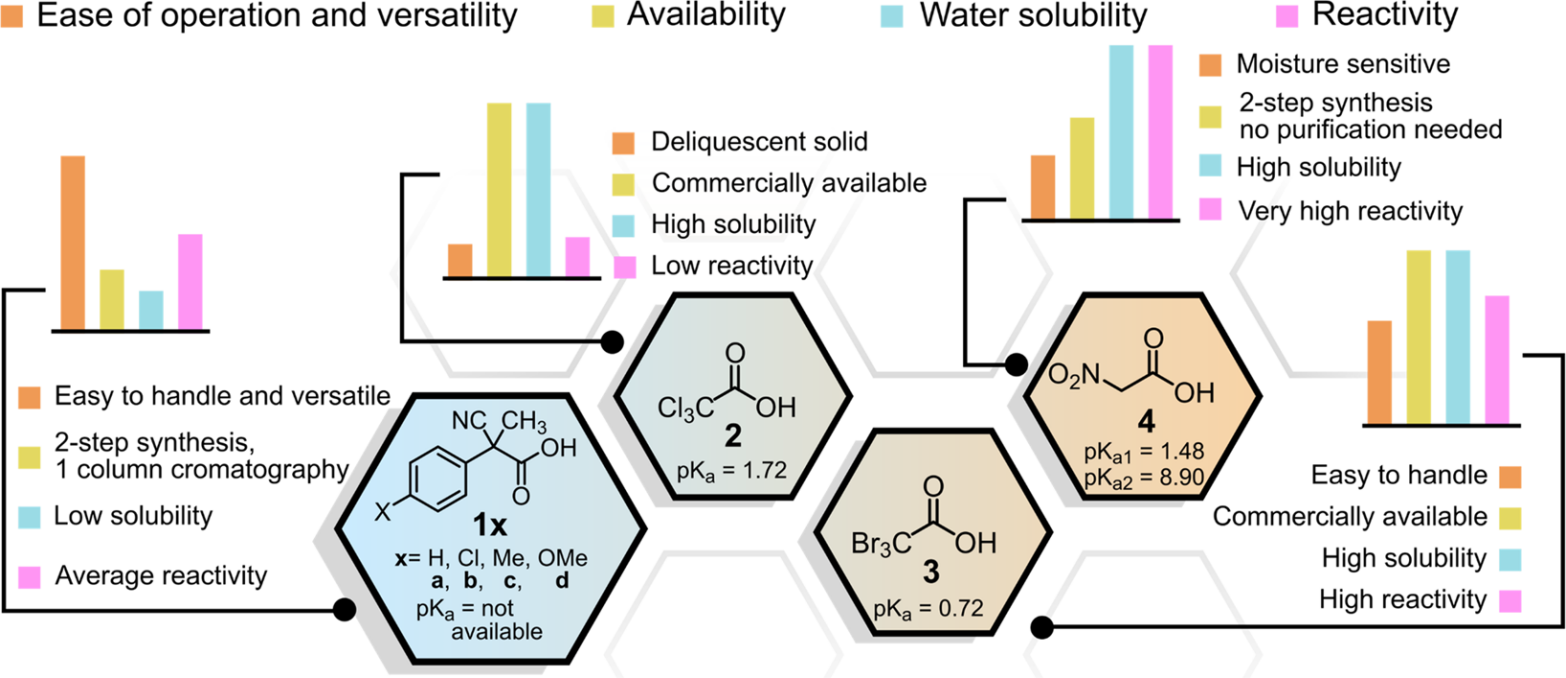
Strengths and weaknesses of the ACAs used so far to drive the operation of dissipative systems.

The ACAs used so far to drive the operation of dissipative systems are reported in Figure 2. It has to be noted that all of them are endowed with electron withdrawing groups in the α position of the carboxylic function, which allow a smooth decarboxylation of the corresponding carboxylates (ACAcbs) under mild conditions by stabilizing the resulting carbanion. 2-Cyano-2-phenylpropanoic acid (**1a**) has been the first one used to drive a dissipative system. It offers some advantages such as high solubility in organic solvents, high stability, and possible derivatization (**1b**, **1c**, and **1d** are easily obtained and used when different operation rates are needed). Furthermore, ACAs **1a**–**d** are easy to handle and weighable, a non-negligible feature when stock solutions with accurately known concentration are needed. Trichloroacetic acid **2** has been also widely used to guide dissipative systems. It has the great advantage of producing chloroform as waste product that in some cases coincides with the solvent in which the system operates. This means that, formally, no waste is produced. However, **2** has the relevant drawback that it is strongly hygroscopic, deliquescent, and hard-to-weigh. In contrast, tribromoacetic acid **3** is not deliquescent albeit decarboxylates more rapidly than **2**. Remarkably, both **2** and **3** have good water solubility and can be used in aqueous solution, as well as nitroacetic acid **4**, another ACA, which has found application in the field. The latter is much more reactive than both **2** and **3** and cannot be easily used in organic solvents where the decarboxylation of the corresponding conjugate base is too fast. Protic solvents generally retard the decarboxylation of ACAcbs by stabilizing them through hydrogen bonding. Conversely, dipolar aprotic solvents accelerate the decarboxylation by separating the ion pair between the ACAcb and its counterion, with consequent destabilization of the anion. Figure 2 summarizes strengths and weaknesses of the different ACAs. It has to be noted that the presence of bases other than the dissipative system (this always occurs in water where pH buffers are needed to set the conditions) necessarily causes a decrease of efficiency of the ACA fuels, providing alternative decarboxylation paths that subtract the acid from the cycle depicted in Figure 1b.

## Dissipative Systems Driven by ACAs

### Molecular Switches and Motors

In this section ACA-driven molecular switches and motors are considered. While switches perform movements with no regard to directionality (no chance of doing “work”), more structured molecular motors are capable of unidirectional motions (in principle, they can do “work”).^14^

**Figure 3:**
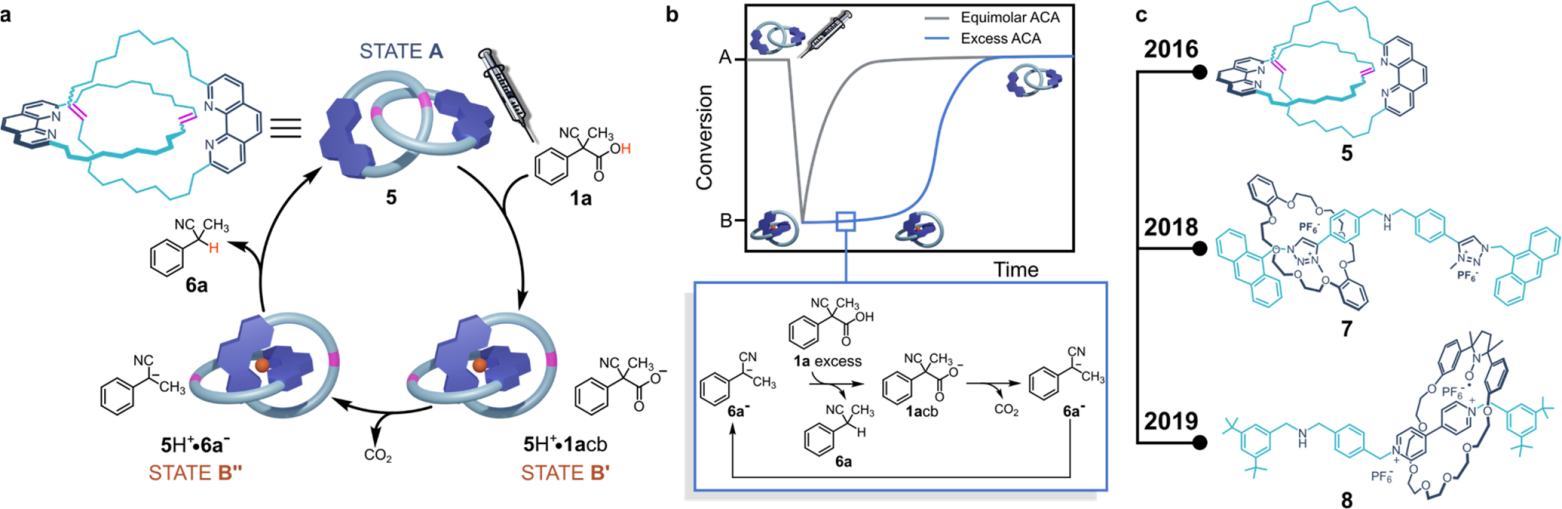
(a) Operation of switch **5** driven by ACA **1a**. (b) **5** is maintained in the protonated state as long as excess **1a** is present. (c) Molecular switches driven by ACAs.

In 2016 for the first time, an ACA, namely, 2-cyano-2-phenylpropanoic acid **1a**, was used to drive the operation of a molecular switch. Sauvage-type [2]-catenane **5** depicted in Figure 3 was shown to perform switching back and forth motions between the resting, neutral state A and the protonated states B′ and B″ under the action of fuel **1a**.^1^

“The unexpected result is sometimes the seed of discovery”. This truthful sentence appears in the first *Accounts of Chemical Research* article by Joseph F. Bunnett,^15^ one of the most illuminated and illuminating physical organic chemists. Being interested in the synthesis of a main-chain polycatenane, we had originally prepared catenane **5** and its Cu^+^ complex in order to achieve polymerization by means of a Grubbs’ catalyst promoted olefin metathesis.^16^ With the aim at carrying out a co-conformational study by ^1^H NMR, we heated a 1,1,2,2-tetrachloroethane-*d*_2_ solution of catenane **5** to 80 °C in a NMR tube and soon realized that it was such as a strong base to be partially protonated (or better to say “deuterated”) by the solvent. The strong basic character^17^ of catenane **5** was the key for the idea of using an ACA to promote a complete back and forth motion cycle of such a molecular switch.

When one molar equivalent of acid **1a** is added to neutral **5** (resting state A, Figure 3a), the immediate proton transfer from **1a** to **5** generates state B′, an ion pair composed of the protonated catenane **5**H^+^ and the conjugate base of **1a**, **1a**cb. A fast decarboxylation follows, which leads to state B″ where a carbanion is intimately bound to the protonated catenane. A final, rate-determining back-proton transfer from **5**H^+^ to the carbanion, a very strong base, restores the neutral, resting state of **5** within a couple of hours under the operative conditions, producing 2-phenylpropanonitrile (**6a**) as the only waste product. For the first time a whole cycle of motion of a molecular switch was driven by one only chemical stimulus without the necessity to involve a counter-stimulus for the back motion. Notably, the duration of the protonated state B″ can be controlled by varying the amount of added fuel. When an excess fuel is added, protonation of the carbanion present in B″ occurs at the expense of the most acidic species, that is the excess acid **1a**. The just formed **1a**cb rapidly decarboxylates to restore state B″, which, consequently, persists in solution as long as the excess fuel is present (Figure 3b). Fuel **1a** and its derivatives **1b**–**d**^2^ were then used as the only stimulus to drive the back and forth motions of other molecular switches (**7** and **8**) based on the acid–base reaction (Figure 3c).^18^ In most cases, the rate-determining step (rds) of the motion cycle of the switch is associated with the decarboxylation step (**1a**-**d**cb → **6a**–**d**^–^); however it may happen, as occurs in the above prototype case,^1,2^ that the rds of the motion cycle is associated with the back proton-transfer from the protonated switch to the carbanion. In any case, since the overall decarboxylation rate of fuels **1a**–**d** increases in the order **1d** < **1c** < **1a**< **1b**, one can choose the proper fuel to control the rate of the motion cycle at will.

Also trichloroacetic acid **2**(19) has been exploited to drive the operation of mechanically interlocked molecules based on the acid–base reaction in solution. In fact, as early as 2012, Takata et al.^20^ showed that the thermal decomposition of the solid trichloroacetate salt of a protonated rotaxane based on the 24-dibenzocrown-8/dibenzylammonium interaction causes the loss of CO_2_, with consequent deprotonation of the rotaxane, shift of the crown ether away from the dibenzylamine station, and production of chloroform.

**Figure 4:**
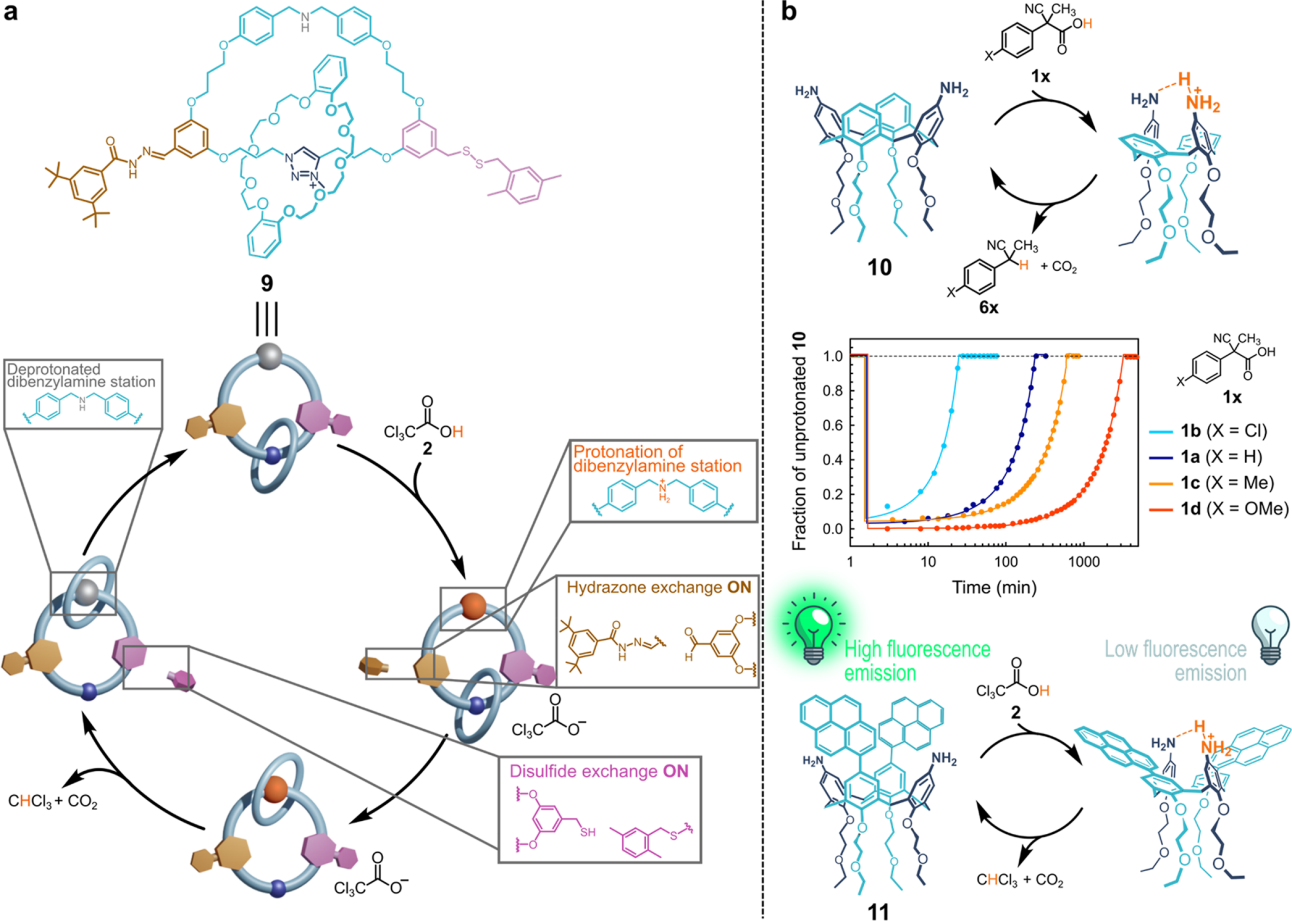
(a) Leigh’s motor **9** driven by ACA **2** (see text). (b) Governing the locked/unlocked states and fluorescence cycles of calix[4]arene scaffolds by ACAs **1a**–**d** and **2**.

More recently, fuel **2** was magisterially used to drive the molecular motor **9** by Leigh and co-workers (Figure 4a).^21^ In this case, the smaller ring in [2]-catenane **9** rotates around the larger one in an oriented fashion due to the presence of a hydrazone- and a disulfide-based stopper that realize an energy ratchet. When the fuel is added to a basic solution of motor **9**, the environment becomes acidic, the hydrazone-based stopper is continuously released and rebound, and the smaller ring has the possibility to reach the protonated secondary amine station on the larger ring (first half rotation, 180°). Upon decarboxylation and chloroform production, the solution becomes basic, and while the hydrazone-based stopper is now stable, the disulfide one starts to be continuously released and re-taken up, giving to the smaller ring the chance to reach the methyl triazolium station, moving from the no more protonated secondary amine (second half rotation, 180°). The same hydrazone–disulfide energy ratchet was then applied to achieve a system in which a biphenyl based crown ether is able to read the chiral information contained in a molecular wire defined as a “molecular tape”.^22^

Both kind of ACAs, fuels **1a**–**d** and **2**, were also applied to finely control the conformation of calix[4]arene cone scaffolds endowed with two amino functions at two opposite positions of the upper rim (see **10** and **11** in Figure 4b). In particular, a time programmable locking/unlocking of the **10** scaffold was achieved using fuels **1a**–**d**.^23^ In the absence of fuel, the structure is free to perform a rapid “fortune teller origami” motion (breathing of the cone calix[4]arene). Addition of the fuel leads to a locked rigid conformation of the scaffold due to the sharing of the proton between the two amine functions. The structure is then unlocked and made free to breathe again after decarboxylation. The duration of the locking/unlocking cycle can be regulated varying both the nature (**1a**–**d**) and the amount of the added fuel. With the same principle, ACA **2** was found to be a convenient fuel to program over time the fluorescence properties of calix[4]arene **11** (Figure 4b), whose pyrene groups are spatially paired in the absence of fuel (fluorescence HIGH) and moved away when the fuel is added (fluorescence LOW). Decarboxylation restores the initial conformation and fluorescence.^24^ The higher the excess of added fuel, the longer the time needed for its dissipation, the longer the duration of the LOW fluorescence state.

**Figure 5:**
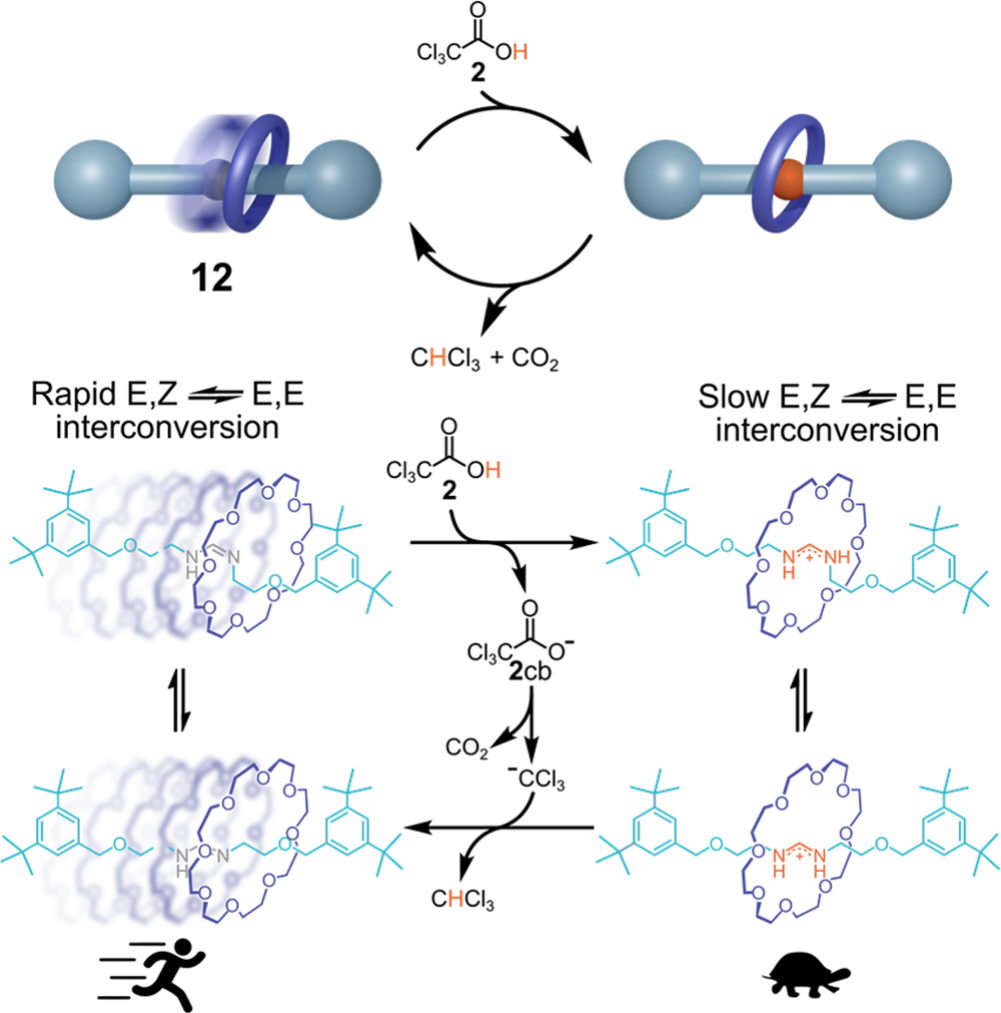
von Delius’ amidine-based [2]-rotaxane under the action of ACA **2**.

The same fuel **2** was also used by von Delius and co-workers to control over the time the configurational stability of the amidine/amidinium function present in the thread of [2]-rotaxane **12** (see Figure 5).^25^ Under basic conditions, the amidine is in its neutral form and a rapid conversion between its *E*–*Z* and *E*–*E* configuration is observed, with the crown-ether wheel moving fast along the thread. Upon addition of **2**, the amidine is protonated to amidinium, which strongly interacts with the crown-wheel. Consequently, the *E*–*Z*/*E*–*E* isomerization is strongly slowed down until decarboxylation of **2**cb is complete. At this point, the amidine core is found again in its neutral form, which allows rapid *E*–*Z*/*E*–*E* isomerization.

### Translocations

**Figure 6:**
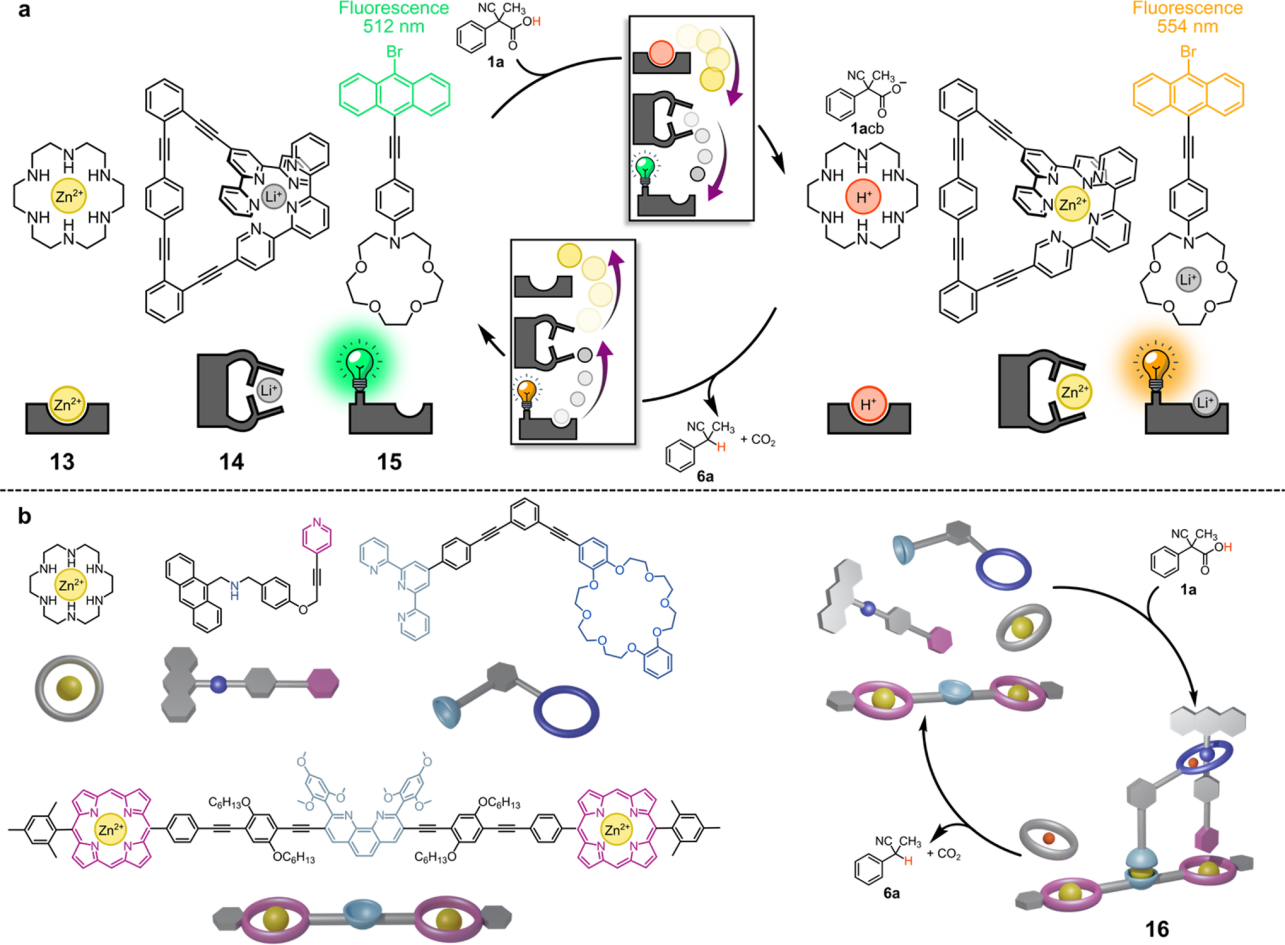
(a) Transient cascade-translocation triggered by ACA **1a** with consequent fluorescence change. (b) A psudorotaxane rotor temporarily assembled from five components by means of **1a**.

Translocation, i.e., the motion of a molecular entity (an ion or a neutral molecule) between two or more sites, is a ubiquitous process in living networks. Since many biological functions such as signal transmission or catalysis often involve translocation, the latter has been mimicked in abiotic systems. Also translocation can be driven in a dissipative fashion using ACAs. Schmittel and co-workers recently showed that the fluorescence emission of a solution containing the hexa-azacrown Zn^2+^ complex **13**, the terpyridine based Li^+^ complex **14**, and the fluorescent functionalized monoazacrown **15** can be controlled employing a cascade of translocation events triggered by **1a** (Figure 6a).^26^ The addition of the fuel causes protonation of the hexa-azacrown ligand, which ejects the Zn^2+^ cation. The latter displaces the Li^+^ cation from the terpyridine ligand, and the Li^+^ cation is in turn captured by the monoazacrown **15**, largely changing its fluorescence properties. From now onward, decarboxylation of **1a**cb takes place, and at the end, the initial state is restored. Thus, both metal cations Zn^2+^ and Li^+^ are translocated between two alternative sites during the fuel decarboxylation process.

The dissipative translocation of the Zn^2+^ cation between hexa-azacrown and terpyridine ligands driven by ACAs **1a** and **1b** has been then studied by XAS-technique showing the robustness of this chemistry.^27^ Such process has been again exploited by Schmittel and co-workers in the dissipative assembly of the five-component pseudorotaxane rotor **16** (Figure 6b).^28^ When fuel **1a** is added to the solution of the five components, **16** is formed with the pseudorotaxane arm moving from one Zn-porphyrin to the other with a frequency of 15.4 kHz at 298 K. The rotor is disassembled when **1a**cb decarboxylates.

Eventually, fuel **2** was exploited to program over time the migration of the Zn^2+^ cation around alternative binding sites present in a tripeptide scaffold.^29^

### Catalysts

Temporal control of catalysis is one of the most demanding goals of chemical research. Here below we will consider two recent examples of dissipative catalysts based on the chemistry discussed in the previous sections, in particular, one involving a molecular switch and the other one a translocation process, that well illustrate the high versatility of the ACA fuel in driving complex systems.

**Figure 7:**
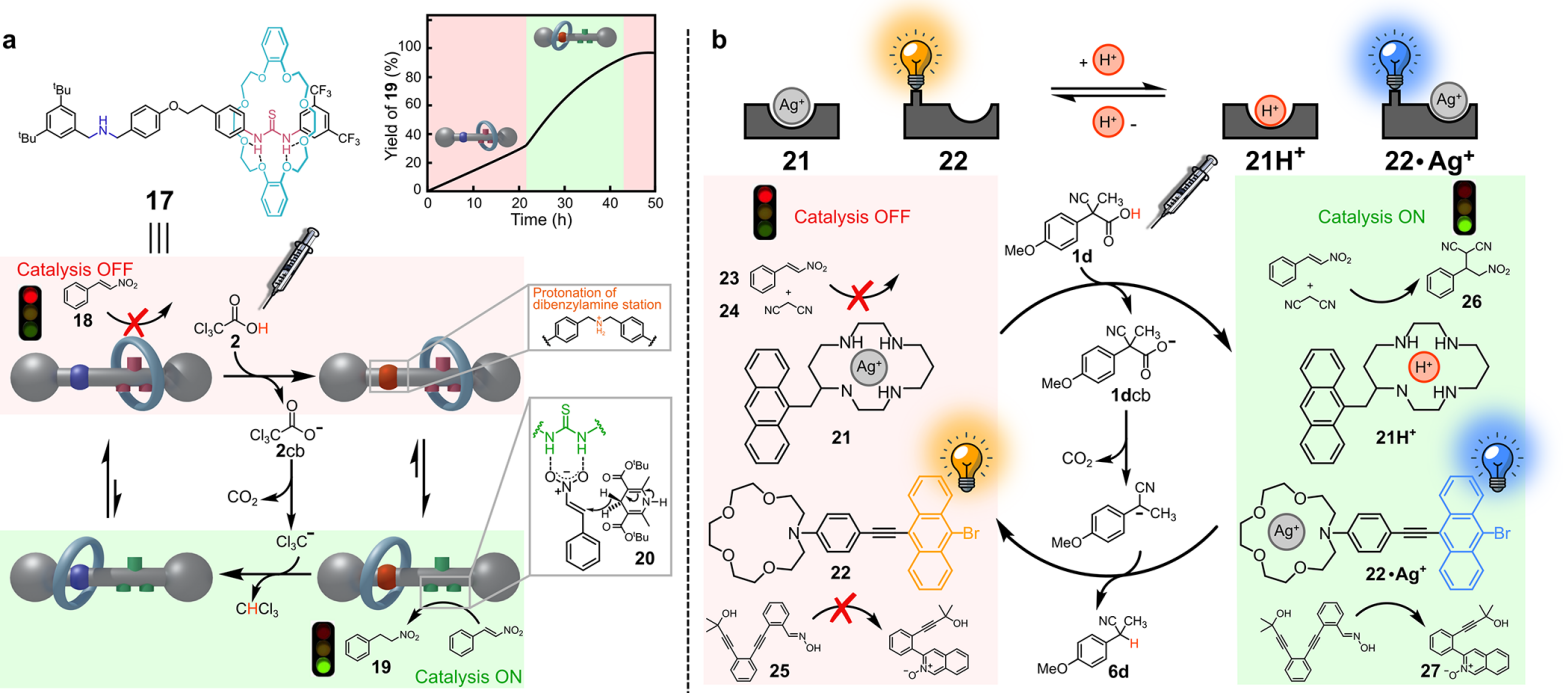
Dissipative catalysis by (a) a molecular switch driven by ACA **2** and (b) a translocation process triggered by ACA **1d** (see text).

Leigh et al.^30^ showed that the catalytic activity of [2]-rotaxane **17**, which is able to catalyze the reduction of nitrostyrene **18** by Hantzch ester **20**, can be controlled at will using ACA **2** (Figure 7a). In the resting state of the switch, the thiourea group, responsible for catalysis, is masked by the crown ether that interacts with it through multiple hydrogen bonds. When **2** is added, the secondary amine is protonated, and consequently, the crown ether shifts to the ammonium station, leaving the thiourea free to exert its catalytic action. The active state of the switch persists as long as the fuel is present, that is until decarboxylation of **2**cb is complete. Then, the crown ether goes back to the thiourea station with interruption of the catalysis. Thus, the duration of the catalyst active state can be varied at will controlling the amount of the added fuel. The larger the amount, the longer the time spent by the switch in the active state.

The dissipative catalytic system recently designed by Schmittel and co-workers consists of the anthracene-appended cyclam **21**, the 9-bromoanthracene-appended aza-crown ether **22**, and AgBF_4_ (see Figure 7b).^31^ In the absence of other additives, the silver cation is complexed in **21** and ligand **22** is found in its free form. Upon adding reagents **23**+**24**, a Michael addition couple, and precursor **25**, nothing happens. However, when fuel **1d** is introduced, **21** is protonated and releases the silver cation that is intercepted by **22**. Now, Michael addition of **23**+**24** leads to **26** due to catalysis by **21**H^+^ as well as cyclization of **25** to isoquinoline-2-oxide **27** due to catalysis by **22**·Ag^+^. Such double catalysis persists as long as the fuel is present and ends when the decarboxylation of **1d**cb is complete. Addition of fresh **1d** allows the catalysis to restart again.

### Dynamic Combinatorial Libraries

ACA fuels can be also used to temporarily alter the composition of Dynamic Combinatorial Libraries (DCLs). DCLs are collections of compounds capable of interconverting each other under equilibrium conditions. The study of such ensembles of compounds (Dynamic Combinatorial Chemistry, DCC) has led in the last quarter of a century to important results in the fields of recognition, self-assembly, catalysis, and, more generally, systems chemistry.^32^ Very recently, we and other groups have explored the possibility of achieving dissipative dynamic combinatorial libraries (DDCLs),^10^ that are dynamic libraries of compounds maintained out-of-equilibrium by the consumption of a fuel. Variation of the nature or the amount of added fuel allows regulation of the duration of the out-of-equilibrium state of the library making available time-control of the library composition and of the related chemical–physical properties.

**Figure 8:**
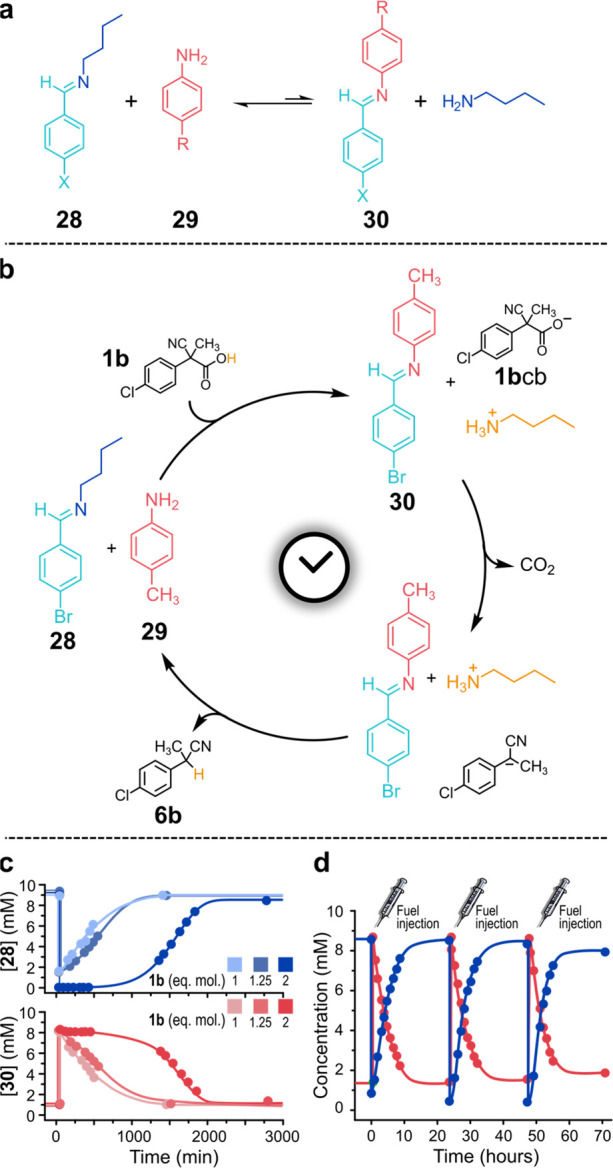
(a) Minimal DCL of imines. (b) A DCL of imines under the action of ACA **1b**. (c) Increasing the amount of added fuel the dissipative state persists longer. (d) Repetitive DCL → DDCL → DCL cycles.

We showed that a minimal DCL of imines can be easily obtained by adding alkylimine **28** to arylamine **29** in a series of solvents including dichloromethane, chloroform, and acetonitrile. In dichloromethane, after 24 h at 25 °C,^4^ the equilibrium is reached and a DCL of two imines and two amines is achieved (Figure 8a, X = Br, R = CH_3_), whose composition is shifted toward the couple **28**+**29**. However, if fuel **1b** is subsequently added, an immediate overturning of the library is observed with a new composition dominated by the alternative arylimine **30** (Figure 8b). The fuel acid has protonated the alkylamine, which is the strongest base in solution, shifting the equilibrium of Figure 8a toward the arylimine. This new DCL, a dissipative DCL (DDCL), is found in an out-of-equilibrium state since **1b**cb starts to lose CO_2_, generating the corresponding carbanion, which immediately deprotonates the alkylammonium ion (Figure 8b). Consequently, due to fast back-transimination, the initial library is restored when decarboxylation of **1b**cb is complete. Remarkably, fuel **1b**, aside from thermodynamically steering the equilibrium between the two imines, also catalyzes the transimination reaction, which allows the rapid reversibility of the DCL. The system was shown to be robust with respect to the nature of the imines/amines, solvents, and temperature. As for time programming, the time spent by the system in the DDCL state (out-of-equilibrium) can be increased at will on increasing the amount of added fuel **1b** (Figure 8c). The higher the excess of added fuel, the longer the time needed for its dissipation (decarboxylation), the longer the duration of the protonated state of the alkylamine. The systems proved highly reversible; three subsequent shots of fuel drove three successive DCL → DDCL → DCL cycles with perfect restoration of the initial conditions each time (Figure 8d). Eventually, more complex DDCLs of imines with reversible and predictable behavior were obtained starting from three or four imines and related amines.^4^

### Host–Guest Systems

Temporal control of the interactions between a host molecule and its guest has been also achieved using ACA fuels. Host–guest chemistry is at the very heart of supramolecular chemistry, thus its time-programmability is particularly desirable.

**Figure 9:**
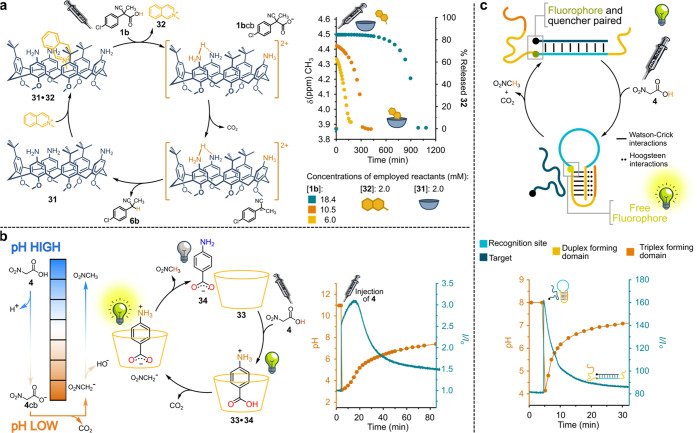
Host–guest interactions temporally driven by ACA fuels. (a) **1b** triggers release–reuptake cycles of **32** from-and-to **31**. (b, c) **4** triggers release-reuptake cycles (b) of **34** from-and-to **33** and (c) of a DNA-target from-and-to a DNA-receptor.

For example, the host–guest interaction between triaminocalix[6]arene **31** and *N*-methylisoquinolinium **32** has been temporally controlled by ACA fuel **1b** (Figure 9a).^33^ When the latter is added to a dichloromethane solution of complex **31**·**32**, due to protonation of the amino groups, **31** is induced to release **32** in the bulk. However, the subsequent decarboxylation of **1b**cb, and following back proton transfer, restore the neutral form of the host, which is now able to re-take up guest **32**. The fraction of released guest and the time spent by it in the bulk solution was regulated by varying the amount of the added fuel.

ACA fuels also proved to be effective tools for time programming host–guest interactions in pure water. We demonstrated that the association between α-cyclodextrin **33** and *p*-aminobenzoic acid **34**, which is strongly pH-sensitive and responsible for an intense fluorescence emission, can be controlled over time taking advantage of nitroacetic acid **4** (Figure 9b).^34^ This acid is the most activated among the ACAs introduced above, and its decarboxylation in aprotic solvents is so fast that its use as a fuel to drive dissipative systems comes out to be very uncomfortable. However, the tendency of **4** to rapidly loose CO_2_ is largely reduced in water where the hydrogen bond interactions with water molecules strongly stabilize carboxylate anion **4**cb. As shown in Figure 9b, addition of excess **4** to a basic solution (pH 11, due to NaOH) of **33** and free **34** causes an immediate pH decrease with a transient uptake of the amino acid into the hydrophobic cavity of the cyclodextrin. Subsequently, decarboxylation of **4**cb takes place with the pH increasing again and consequent release of **34**. More precisely, since the affinity of the different protonation states of **34** for **33** increases in the order **34**^–^ ≪ **34**^+^ < **34**, at the addition of fuel **4**, an initial immediate increase of the fluorescence is observed, followed by a further and slower increase (until the highest concentration of the zwitterion is reached, highest affinity between the amino acid and **33**), and a final and definite decrease.

Subsequently, the same concept has been employed to drive the operation of a DNA-based nanodevice undergoing duplex–triplex transitions at acidic pH.^3^ The pH-responsiveness is due to a number of cytosine bases in the engineered oligonucleotide sequences, which form an intramolecular triplex at low pH by means of Hoogsteen interactions between complementary cytosine–guanine pairs and cytosineH^+^. Fuel **4** was used to program over time the release of a small DNA strand from a DNA-based receptor (Figure 9c). The receptor is a stem–loop sequence, with the stem containing a cytosine-rich pH-sensitive sequence and the loop including the sequence for binding of the target (the recognition site). At slightly basic pH, the target is bound to the recognition site, while at lower pH, the stem–loop, stabilized by the Hoogsteen interactions, hampers the association between target and receptor. When fuel **4** is added to a basic solution of the target–receptor complex, first the target is immediately released by the receptor, then, due to decarboxylation of **4**cb, slowly re-taken up, as demonstrated by fluorescence monitoring. The percentage of DNA-target transiently released in the bulk by the receptor (from 10 to 90%) can be controlled by varying the amount of added fuel.

### Molecular Cages

**Figure 10:**
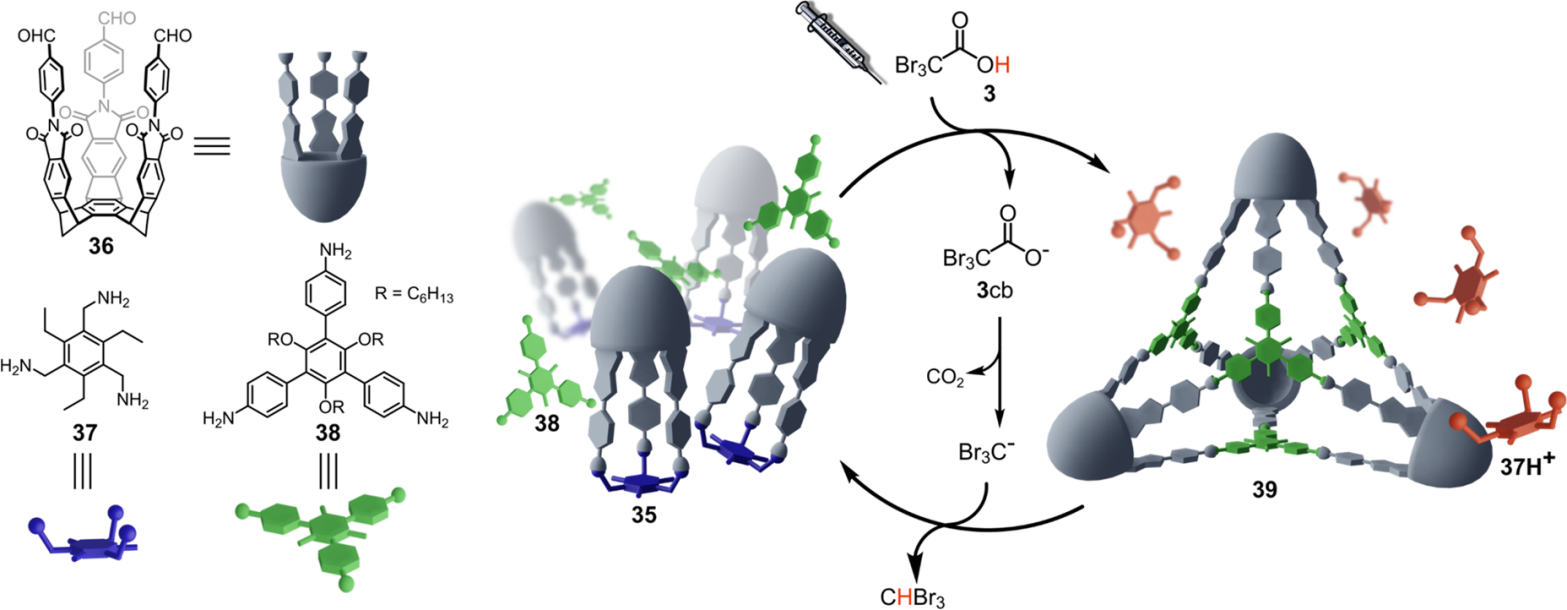
Dissipative formation of capsule **39** driven by ACA **2**.

A recent report by Badjic and co-workers^35^ shows that tribromoacetic acid **3**, can be employed to control over time the assembly of a nanosized cage (Figure 10). The small imine based cage **35** is the triply condensed product of the reaction between the basket-shaped *tris*-aldehyde **36** and *tris*-amine **37**. When **35**, *tris*-aniline **38** and **37** are added in dichloromethane in the presence of a proper amount of trifluoroacetic acid, a heterogeneous mixture is obtained where **35** and **38** are dissolved in solution and **37**, present in its different protonated forms (from mono- to triprotonated), is found as a precipitate. Addition of fuel **3** causes an imine exchange of the same nature of that previously described, and the new nanosized cage **39** is transiently generated at the expense of **35**. The tetrameric capsule **39** persists in solution until decarboxylation of **3**cb is complete. Then, it is disassembled to be replaced by the smaller **35** again.

### Smart Materials

**Figure 11:**
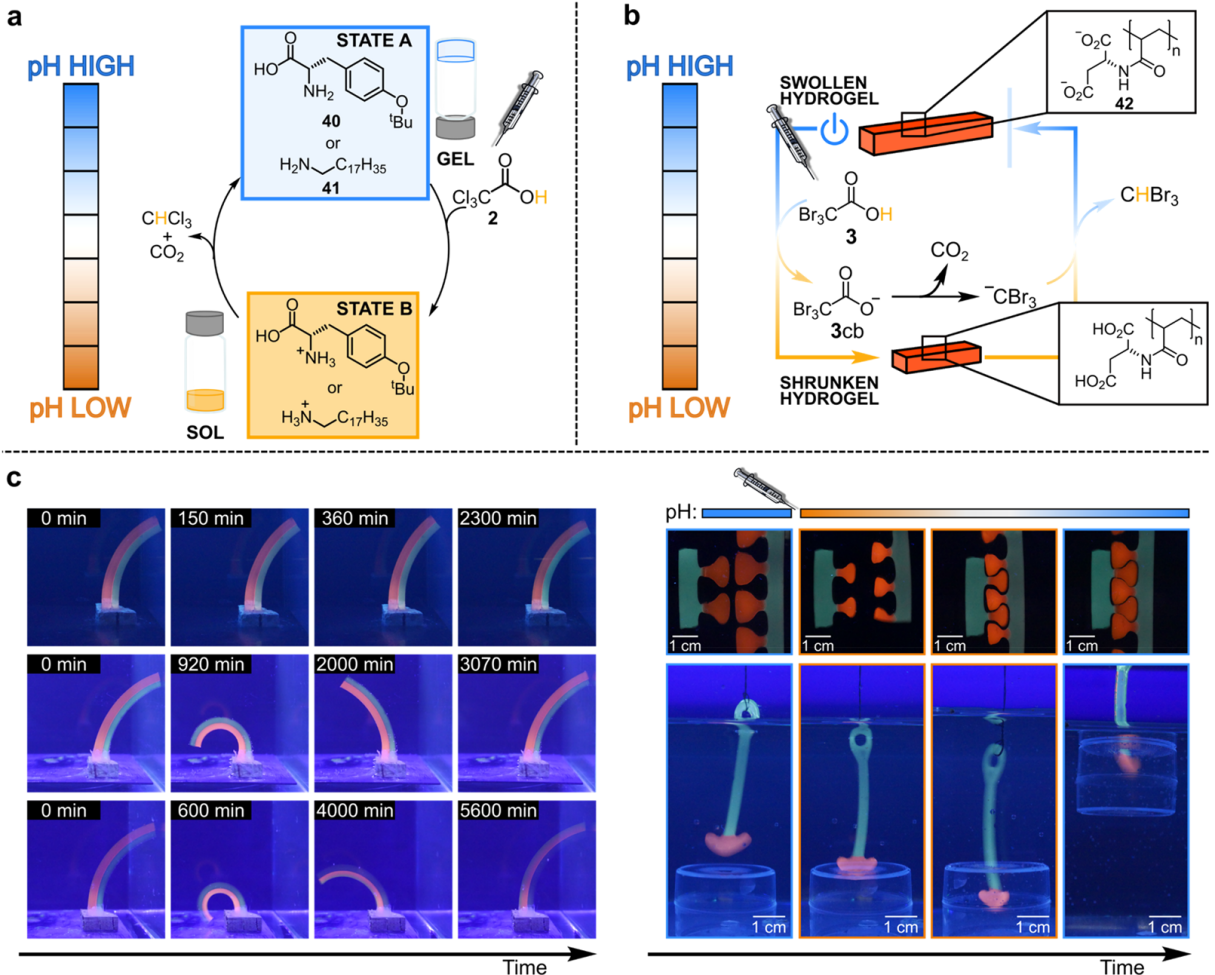
(a) Gel–sol–gel transitions driven by **2**. (b) Temporal control of hydrogel sizes by **3** in water/DMSO. (c) Related applications: transient bending (increasing **3** from top to bottom), puzzle pieces interlocking, object lifting.

ACA fuels were also employed to drive the operation of pH-responsive smart materials. Compared to the widely used clock reactions or pH-feedback systems based on enzymatic reaction networks,^36^ the ACA method is based on a one-component fuel and requires simple operative conditions, a key feature for application-oriented uses. Quintard and co-workers pioneered the use of **2** to achieve sol–gel–sol transitions involving a pH-responsive organogelator.^37^ Then, the same group studied the **2**-driven transient degelation of organogelators **40** and **41**, achieving complementary gel–sol–gel transitions.^38^ Addition of **2** to the neutral organogel (state A, Figure 11a), causes protonation of the organogelator amine moieties, which results in the breakup of the self-assembled state, leading to a clear solution (state B). As the following decarboxylation takes place, the system reverts to the initial gelated state A. The higher the amount of added **2**, the longer the duration of state B. Such an easy-to-handle programmable degelation was applied to develop remoldable objects, erasing inks, and transient electrical junctions.

ACA **3** was instead employed to program over time the autonomous operation of aspartic acid-based pH-responsive hydrogels (**42**, Figure 11b).^39^ At neutral pH such hydrogels are swollen because of the electrostatic repulsion among the deprotonated carboxylate groups of **42**. Addition of **3** lowers the pH, leading to the protonation of the carboxylates which causes gel shrinking. Eventually, after decarboxylation, the pH is reset to the initial value inducing the hydrogel reswelling. Such transient (de)swelling can be programmed at will, in terms of amplitude and duration, by varying the amount of added fuel, as demonstrated with fuel-dependent actuation of hydrogel bilayers (Figure 11c, left). Moreover, **3** was exploited to achieve more complex tasks, such as interlocking of puzzle pieces and lifting of objects (Figure 11c, right).

Eventually, bilayers capable of self-regulation through chemo-mechanical feedback were developed, by coupling **3**-driven actuation with localized ammonia production by means of mechanically activated urea/urease reaction.^36^

## Conclusions

In the previous sections we have shown that the ACA fuels have found application in different fields of dissipative systems. Such generality is due to the simplicity of the operation principle underlying the ACA fuels, which is based on two simple reactions: (i) the acid–base reactions involving the operating system and (ii) the decarboxylation of the fuel. Both reactions are clean and intrinsically robust in regards to experimental conditions, although variations of the decarboxylation rate are observed when solvent, temperature, nature of the ACA, and nature of the basic function present in the system are changed. Nevertheless, such variations are highly welcome since they allow time-programmability of dissipative systems. We have indeed shown that the duration of the dissipative state of the system can be easily regulated at will by changing the nature and the amounts of the ACA fuels. Furthermore, ACA fuels are commercially available or, if not, very easy to prepare, and the majority of them are easy to handle with no particular precaution required.

Yet, some drawbacks have to be considered when ACA fuels are used. Waste production (RH besides CO_2_, see Figure 1b) can negatively affect the system operation; furthermore, despite ACAs having proven to be highly versatile in organic media, this feature is lacking in pure water (acid **4** is the only ACA used so far, whose decarboxylation rate is high enough for a convenient use in pure water). The design of new ACAs capable of solving both the above issues may open new scenarios for the application of such fuels, providing a simple tool to program over time the operation of pH responsive molecular systems and smart materials in water.

Overall, it is expected that, in the near future, the use of ACAs as chemical fuels for driving the operation of dissipative systems based on the acid–base reaction will become increasingly widespread.
